# Effects of social media use on depressive symptoms among Chinese university students: the mediating roles of self-esteem and social support

**DOI:** 10.3389/fpsyg.2026.1769765

**Published:** 2026-03-12

**Authors:** Li Ma, Yujia Li

**Affiliations:** School of Humanities and Social Science, Taiyuan University of Science and Technology, Shanxi, China

**Keywords:** Depressive tendencies, self-esteem, social media use, social support, university students

## Abstract

Social media use is pervasive among university students and young adults; however, its psychological implications remain debated. This study examined whether self-esteem and perceived social support mediated the association between social media use and depressive tendency. A cluster sample of 635 Chinese undergraduates from universities in Guangdong Province was recruited, yielding 600 valid responses (97.7%). Participants completed standardized scales including the Bergen Social Media Addiction Scale, the Rosenberg Self-Esteem Scale, the Multidimensional Scale of Perceived Social Support, and the Zung Self-Rating Depression Scale. Structural equation modeling with 5,000 bootstrap resamples tested direct and indirect effects. Social media use was positively associated with depressive symptoms and negatively with self-esteem and social support. Both self-esteem and social support mediated this relationship, with stronger effects for self-esteem. Higher levels of social media use were associated with higher depressive symptoms, both directly and indirectly through reduced self-esteem and perceived social support, with self-esteem playing a stronger mediating role.

## 1 Introduction

Depression is a common emotional disorder among college students (Ibrahim et al., 2013). Epidemiological evidence indicates a high and increasing prevalence of depressive symptoms in university students. A large meta-analysis reported a detection rate of depressive symptoms as high as 38.7% (Lin et al., 2025), with some individuals experiencing significant difficulties in academic performance, interpersonal relationships, and emotional regulation. Persistent depression not only reduces learning efficiency and social interaction but may also lead to self-harm and suicidal behaviors (LeMoult and Gotlib, 2019; Ordaz et al., 2018). Consequently, it has been recognized by the World Health Organization as a major public health issue affecting young people's development and social adaptation (Rogers et al., 2018). Depressive tendency is considered a prodromal state of depression, referring to individuals who have not met clinical diagnostic criteria but exhibit a certain level of depressive symptoms. Studies suggest that depressive tendencies are prevalent in university students and significantly increase the risk of future major depressive disorder and other mental health problems (Wesselhoeft et al., 2013), with some surveys reporting nearly 50% detection rate among college students (Luo et al., 2024). Therefore, early identification and intervention for depressive tendencies have important practical implications.

The widespread use of mobile internet has profoundly reshaped the developmental environment of university students (Sánchez-Fernández and Borda-Mas, 2023). According to the 52nd “Statistical Report on Internet Development in China,” instant messaging and short video usage rates among Chinese internet users reached 97.1% and 95.2%, respectively (China Internet Network Information Center, 2023). While social media provides university students with instant connection, emotional expression, and information access, it also introduces new stressors such as fear of missing out (FOMO), evaluation dependence, and social comparison (Tandon et al., 2021). Empirical studies have shown that overall usage, nighttime usage, and emotional investment in social media are all associated with higher depressive symptoms and lower self-esteem (Woods and Scott, 2016). Reviews also indicate that although the overall effect size between social media use and self-esteem is relatively small, it is more pronounced in problematic use or specific platforms (e.g., Instagram) and shows significant individual variability (Cingel et al., 2022). These findings suggest that the relationship between social media use and depression is not unidirectional but may be regulated by mediating mechanisms.

Regarding potential mechanisms, self-esteem and social support are considered two key psychological pathways. First, idealized presentations on social media can trigger upward social comparisons, eroding self-worth and increasing the risk of depression. Domestic studies have shown that social networking site use can reduce university students' self-esteem through upward comparisons, which in turn predicts depressive symptoms, forming a chain mediation pathway of “use → comparison → self-esteem → depression” (Niu et al., 2016). Second, passive browsing on social media may weaken real-life social connections and perceived social support. Previous longitudinal studies have found that passive Facebook use decreases both online and offline social support, which subsequently predicts higher depressive symptoms 6 months later (Park et al., 2016). Impaired self-esteem and diminished social support may serve as two parallel mediating pathways through which social media use affects depressive tendencies.

Despite the growing body of research, three limitations remain. First, existing studies often focus on the direct association between social media use and depression, lacking systematic examination of the parallel mediating roles of self-esteem and social support. Second, studies frequently mix indicators such as overall usage time and problematic use, leading to context-dependent effect sizes and platform-specific differences. Third, in young adult samples within the Chinese cultural context, evidence for an integrated model of “social media → self-esteem/social support → depressive tendency” remains limited. Addressing these issues can help explain individual differences at the mechanistic level and provide actionable guidance for school-based psychological education and social interventions.

**The purpose of this study was to examine the impact of social media use on depressive tendencies in university students and to investigate the mediating roles of self-esteem and social support**. We employed structural equation modeling (SEM) to characterize the overall relationships among variables and used the Bootstrap method to assess the robustness of indirect effects, aiming to address three core questions: (1) Does social media use significantly predict depressive tendencies in university students? (2) Do self-esteem and social support function as independent parallel mediators? (3) What are the relative contributions of these two psychological pathways? The expected contribution of this study is to provide mechanistic evidence for the relationship between social media use and depressive tendencies in Chinese university students, clarifying the roles of impaired self-esteem and reduced social support, and informing the identification of high-risk groups as well as targeted school and social interventions.

## 2 Materials and methods

### 2.1 Participants

This study employed a cluster sampling method, randomly selecting 635 undergraduate students from two universities in Guangdong Province in South China. All participants provided informed consent and participated voluntarily. Inclusion criteria were: (1) age between 18 and 22 years; (2) ability to complete online questionnaires independently; and (3) no history of severe psychiatric disorders or cognitive impairments. After excluding incomplete or invalid responses, 600 valid questionnaires were obtained, yielding an effective response rate of 97.7%. The survey was conducted by the author's team at two collaborating universities in Guangdong Province.

### 2.2 Social media use

The Bergen Social Media Addiction Scale (BSMAS) was used to assess individuals' social media use. Although originally developed to measure problematic use, it has been widely applied as an indicator of social media engagement intensity among university students and young adults (Bottaro et al., 2025). The scale consists of six items reflecting core characteristics such as addictive use, tolerance, and withdrawal. Each item is rated on a 5-point Likert scale (1 = never, 5 = always), with total scores ranging from 6 to 30; higher scores indicate more frequent or dependent social media use. The BSMAS has demonstrated good reliability and validity in Chinese young adult populations (Leung et al., 2020). In the present study, this measure was used to operationalize problematic patterns of social media use rather than normative or purely time-based usage.

### 2.3 Self-esteem and social support

Self-esteem was assessed using the Rosenberg Self-Esteem Scale (RSES), which includes 10 items, five of which are reverse-scored. Items are rated on a 4-point Likert scale (1 = strongly disagree, 4 = strongly agree), with total scores ranging from 10 to 40; higher scores indicate higher self-esteem. The scale demonstrates good internal consistency in Chinese young adult populations (Jiang et al., 2023).

Social support was measured using the Multidimensional Scale of Perceived Social Support (MSPSS), which contains 12 items assessing support from family, friends, and significant others. Items are rated on a 7-point Likert scale (1 = strongly disagree, 7 = strongly agree), with higher scores indicating greater perceived social support (Dambi et al., 2018).

### 2.4 Depressive tendency

Depressive tendency was assessed using the Zung Self-Rating Depression Scale (SDS), which consists of 20 items rated on a 4-point Likert scale (1 = rarely or none of the time, 4 = most or all of the time). Following standard scoring procedures, total SDS scores were calculated and standardized, with higher scores indicating more severe depressive tendencies. The SDS is widely used among Chinese college students (Zung, 1965).

### 2.5 Statistical analysis

Cronbach's α coefficients were used to assess the internal consistency of each scale. Descriptive statistics and Pearson correlation analyses were conducted to examine the distributions and linear relationships among social media use, self-esteem, social support, and depressive tendency. Mediation effects were tested using structural equation modeling (SEM) with the lavaan package in R 4.4.3, specifying a multiple mediation model of “social media use → self-esteem/social support → depressive tendency.” To improve the robustness of effect estimates, the Bootstrapping method with 5,000 resamples was employed to calculate 95% confidence intervals for indirect effects. Mediation was considered significant if the confidence interval did not include zero.

## 3 Results

### 3.1 Descriptive statistics of variables

The mean scores were as follows: social media use (BSMAS) 20.14 ± 4.91, self-esteem (RSES) 30.85 ± 6.35, social support (MSPSS) 58.62 ± 11.02, and depressive tendency (SDS) 54.22 ± 11.45. All scales demonstrated good internal consistency (Cronbach's α ranging from 0.875 to 0.915). Correlation analyses indicated that social media use was positively associated with depressive tendency (*r* = 0.43) and negatively associated with self-esteem (*r* = −0.36) and social support (*r* = −0.29). Self-esteem and social support were both negatively correlated with depressive tendency (*r* = −0.39 and *r* = −0.26, respectively), and self-esteem was positively correlated with social support (*r* = 0.32) (Table 1).

**Table 1:** Descriptive statistics of variables.

**Variable**	**BSMAS**	**RSES**	**MSPSS**	**SDS**
BSMAS	1			
RSES	−0.360^*^	1		
MSPSS	−0.290^**^	0.320^**^	1	
SDS	0.43^**^	−0.390^**^	−0.260^*^	1
M	20.14	30.85	58.62	54.22
SD	4.91	6.35	11.02	11.45
Cronbach α	0.885	0.875	0.915	0.895

^*^p < 0.05, ^**^p < 0.01, ^***^p < 0.001.

### 3.2 Mediation analysis

**Figure 1:**
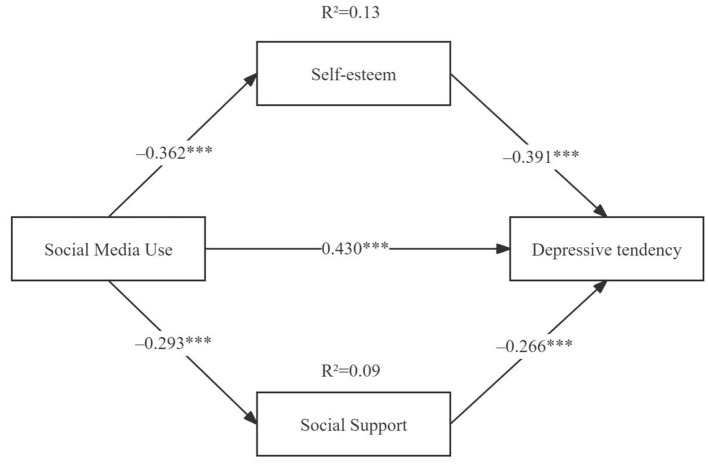
Mediation analysis. *p < 0.05, **p < 0.01, ***p < 0.001.

Mediation analysis indicated that social media use significantly negatively predicted self-esteem [*a1* = −0.362, *t* = −8.419, 95% CI = (−0.446, −0.278)] and social support [*a*_2_ = −0.293, *t* = −7.513, 95 CI = (−0.369, −0.217)]. Self-esteem [*b1* = −0.391, *t* = −10.263, 95 CI = (−0.465, −0.317)] and social support [*b*_2_ = −0.266, *t* = −7.429, 95 CI = (−0.335, −0.197)] were both significant negative predictors of depressive tendency. The direct effect of social media use on depressive tendency remained significant [*c*′ = 0.430, *t* = 8.600, 95 CI = (0.332, 0.528)], indicating partial mediation (Figure 1 and Table 2).

**Table 2:** Mediation analysis.

**Path**	**β**	***SE***	***t***	***95 % CI***
*a1* Social media use → self-esteem	−0.362	0.043	−8.419	[−0.446, −0.278]
*a_2_* Social media use → social support	−0.293	0.039	−7.513	[−0.369, −0.217]
*b1* Self-esteem → depressive tendency	−0.391	0.038	−10.263	[−0.465, −0.317]
*b_2_* Social support → depressive tendency	−0.266	0.035	−7.429	[−0.335, −0.197]
*c'* Direct effect	0.430	0.050	8.600	[0.332, 0.528]

Bootstrapping with 5,000 resamples was used to further examine the mediation effects. The total indirect effect was 0.253 [95 CI = (0.226, 0.358)], accounting for 58.3% of the total effect. Specifically, the indirect effect via self-esteem was 0.143 [95 CI = (0.102, 0.183)], contributing 40.3%, and via social support was 0.075 [95 CI = (0.044, 0.112)], contributing 18.0%. Overall, the results suggest that social media use indirectly increases depressive tendency by reducing self-esteem and social support, with self-esteem showing a stronger mediating role. The mediation model demonstrated good fit (χ^2^/df = 1.8, CFI = 0.97, RMSEA = 0.045) (Table 3).

**Table 3:** Mediation effect test.

**Effect type**	**β**	***SE***	***95 % CI***	***Proportion (%)***
Direct effect	0.421	0.048	[0.332, 0.528]	41.70%
Total indirect effect	0.253	0.022	[0.226, 0.358]	58.30%
Self-esteem mediation	0.143	0.021	[0.102, 0.183]	40.30%
Social support mediation	0.075	0.022	[0.044, 0.112]	18.00%

^*^p < 0.05, ^**^p < 0.01, ^***^p < 0.001.

## 4 Discussion

Based on a sample of 600 Chinese college students, this study systematically examined the relationships among social media use, self-esteem, social support, and depressive tendency. The results indicated that social media use was significantly positively correlated with depressive tendency (*r* = 0.43) and exerted partial mediation through self-esteem and social support: the indirect effect via self-esteem (β = 0.140) accounted for 40.3% of the total effect, and the indirect effect via social support (β = 0.075) accounted for 18.0%, together contributing over half of the total effect (58.3%). The direct effect remained significant (β = 0.421), suggesting that social media influences depressive tendency both directly and indirectly through reductions in self-esteem and perceived social support. The model demonstrated good fit (χ^2^/ df = 1.8, CFI = 0.97, RMSEA = 0.045), further supporting the robustness of the findings. These findings extend existing media psychology frameworks by clarifying the parallel psychological mechanisms linking problematic social media engagement to depressive risk among Chinese university students.

The study further revealed a dual psychological mechanism through which social media use affects depressive tendency. First, the self-esteem pathway plays a central role. Frequent social media use is often accompanied by continuous exposure to idealized content and upward social comparisons, which gradually undermines individuals' self-worth, leading to reduced self-esteem and, consequently, increased depressive risk (Harris and Orth, 2020; Mann and Blumberg, 2022). This “social comparison → diminished self-esteem → heightened depression” chain is highly consistent with our findings, and the indirect effect through self-esteem accounts for 40.3% of the total effect, indicating its key mediating role in the association between social media use and depressive tendency. Second, the social support pathway should not be overlooked. Excessive reliance on online interactions often reduces real-life communication and face-to-face relationship investment via a “time displacement effect,” thereby weakening individuals' perception of reliable social support and increasing psychological vulnerability (Naslund et al., 2016; Shah et al., 2019). In this study, the indirect effect through social support accounted for 18.0% of the total effect. Although smaller than the self-esteem pathway, it still played a significant role. Together, reductions in self-esteem and social support constitute a dual-track mechanism by which social media use influences depressive tendency, with self-esteem exerting a more prominent effect. This finding not only supplements existing theoretical frameworks but also suggests that interventions should prioritize self-esteem enhancement while simultaneously strengthening social support networks to provide dual protection for university students' mental health.

Our findings align with the general trends reported in previous domestic and international studies. Prior literature consistently shows that social media use is significantly associated with depression, anxiety, and reduced self-esteem (Ahmed et al., 2024; Alonzo et al., 2021; Cingel et al., 2022). This study further revealed that self-esteem serves as the primary mediating mechanism, consistent with the theoretical pathway of “social comparison → diminished self-worth → emotional distress” (Andreassen et al., 2017; Pantic, 2014; Pop et al., 2022). Idealized displays and upward social comparisons on social media platforms can undermine university students' self-evaluation, thereby increasing depressive risk (Lin et al., 2016; Lopes et al., 2022). Meanwhile, the mediating role of social support should not be ignored. Passive browsing and excessive use may reduce real-life interactions and relationship quality, lowering university students' perception of reliable social support. This mechanism was confirmed in the present study, with a relatively stronger mediating effect compared to previous reports that found smaller or inconsistent effects (Coyne et al., 2019).

This study makes several important contributions. First, it clarifies the parallel mediating roles of self-esteem and social support, providing an integrated psychological explanation for how social media use relates to depressive tendency among university students. Second, evidence from South China enriches the geographical representation of the literature by adding data from an underrepresented cultural context. Finally, the use of structural equation modeling with 5,000 bootstrap resamples strengthens the robustness and reliability of the mediation findings.

Despite these meaningful findings, several limitations should be noted. First, the cross-sectional design limits causal inference, and reverse causality or third-variable effects cannot be ruled out. For instance, individuals with elevated depressive symptoms may increase social media use as a maladaptive coping strategy, thereby creating a reciprocal feedback loop. Longitudinal evidence has reported bidirectional associations between social media use and depressive symptoms in adolescents and emerging adults (Ren et al., 2026), and recent longitudinal research among Chinese university students also supports reciprocal links between problematic social media use and depression (Luo et al., 2025). Second, the sample was drawn from two universities in Guangdong Province, limiting generalizability to other regions or age groups. Third, the study relied primarily on self-report questionnaires, which may be subject to social desirability or recall biases. Future research should employ longitudinal or experimental designs, incorporate multi-source and objective data (e.g., passive usage logs, sleep tracking), and further examine potential mediating and moderating factors to more comprehensively elucidate causal pathways and individual differences in the relationship between social media use and depression.

## 5 Conclusion

This study suggests that higher levels of social media use are associated with higher depressive symptoms among university students in Guangdong Province, both directly and indirectly through reduced self-esteem and perceived social support. Self-esteem emerged as the stronger mediating pathway. These findings underscore the importance of interventions aimed at enhancing self-esteem and strengthening social support to reduce the mental health risks linked to problematic social media engagement. Moreover, by providing evidence from South China, this study contributes to improving the cross-cultural generalizability of psychological mediation models in digital mental health research.

## Data Availability

The raw data supporting the conclusions of this article will be made available by the authors, without undue reservation.
